# Clonal serotype 1c multidrug-resistant *Shigella flexneri* detected in multiple institutions by sentinel-site sequencing

**DOI:** 10.3389/fmed.2022.964640

**Published:** 2022-08-01

**Authors:** Karrie K. K. Ko, Joash Jun Keat Chu, Kar Mun Lim, Hatairat Yingtaweesittikul, Wenjie Huang, Shireen Yan Ling Tan, Kenneth Choon Meng Goh, Si Huei Tan, Tong Yong Ng, Matthias Maiwald, Jonathan Wei Zhong Chia, Delphine Yanhong Cao, Yen Ee Tan, James Heng Chiak Sim, Tse Hsien Koh, Niranjan Nagarajan, Chayaporn Suphavilai

**Affiliations:** ^1^Department of Microbiology, Singapore General Hospital, Singapore, Singapore; ^2^Department of Molecular Pathology, Singapore General Hospital, Singapore, Singapore; ^3^Genome Institute of Singapore, Agency for Science, Technology and Research, Singapore, Singapore; ^4^Yong Loo Lin School of Medicine, National University of Singapore, Singapore, Singapore; ^5^Duke-NUS Medical School, Singapore, Singapore; ^6^Advanced Research Center for Computational Simulation, Chiang Mai University, Chiang Mai, Thailand; ^7^Department of Laboratory Medicine, Changi General Hospital, Singapore, Singapore; ^8^Department of Pathology, Sengkang General Hospital, Singapore, Singapore; ^9^Department of Pathology and Laboratory Medicine, KK Women's and Children's Hospital, Singapore, Singapore; ^10^Department of Laboratory Medicine, Tan Tock Seng Hospital, Singapore, Singapore

**Keywords:** *Shigella flexneri*, multidrug-resistant (MDR), surveillance, whole-genome sequence (WGS), outbreak

## Abstract

*Shigella flexneri* is a major diarrhoeal pathogen, and the emergence of multidrug-resistant *S. flexneri* is of public health concern. We report the detection of a clonal cluster of multidrug-resistant serotype 1c (7a) *S. flexneri* in Singapore in April 2022. Long-read whole-genome sequence analysis found five *S. flexneri* isolates to be clonal and harboring the extended-spectrum β-lactamases *bla*_CTX−M−15_ and *bla*_TEM−1_. The isolates were phenotypically resistant to ceftriaxone and had intermediate susceptibility to ciprofloxacin. The *S. flexneri* clonal cluster was first detected in a tertiary hospital diagnostic laboratory (sentinel-site), to which the *S. flexneri* isolates were sent from other hospitals for routine serogrouping. Long-read whole-genome sequence analysis was performed in the sentinel-site near real-time in view of the unusually high number of *S. flexneri* isolates received within a short time frame. This study demonstrates that near real-time sentinel-site sequence-based surveillance of convenience samples can detect possible clonal outbreak clusters and may provide alerts useful for public health mitigations at the earliest possible opportunity.

## Introduction

*Shigella* is a major diarrhoeal pathogen and the second leading cause of diarrhoeal mortality globally (1). The pathogen is transmitted through the fecal-oral route, and adults can be infected by as few as 10 bacilli (2), making it highly transmissible. The clinical manifestations of *Shigella* infection range from self-limited diarrhea to fulminant dysentery and life-threatening invasive systemic infections. Shigellosis is estimated to cause more than 200,000 deaths annually, and the greatest burden of morbidity and mortality is among children below the age of five in lower-middle-income countries, as well as adults aged 70 and older (1, 3, 4).

Among the four *Shigella* subgroups (*Shigella dysenteriae, Shigella flexneri, Shigella boydii*, and *Shigella sonnei*), *S. flexneri* and *S. sonnei* cause the majority of laboratory-confirmed *Shigella* infections in developing countries and developed countries, respectively (5, 6). *Shigella flexneri* thus incurs a substantial disease burden among disadvantaged populations worldwide. Further complicating the control of *S. flexneri* infections is the increasing resistance to third-generation cephalosporins in Asia (7–11), and the emergence and dissemination of multidrug-resistant (MDR) *S. flexneri* strains, particularly among men who have sex with men (MSM) (12).

The rise of resistance against third-generation cephalosporins, ciprofloxacin, and azithromycin, coupled with the highly transmissible nature of *S. flexneri*, therefore represents a significant public health threat. Fast and accurate detection of *S. flexneri* clusters enables rapid investigation of outbreak sources and early mitigating actions, thereby enabling public health actions at the earliest opportunity.

Traditional diarrhoeal illness surveillance relies on syndrome-based as well as laboratory-based notification of public health authorities. However, jurisdictions differ in terms of notification requirements for *Shigella* infections. For instance, the UK Health Security Agency requires healthcare practitioners to notify the relevant public health agencies when a case of *Shigella* infection is detected (13), whereas some other jurisdictions do not (14). Where notification is not legally required, the public health system relies on astute individual healthcare practitioners to make the decision to notify the public health agency when a *Shigella* cluster is suspected. Such reliance on individual decisions in a complex healthcare system carries disadvantages. Most clinicians will only be aware of cases within their area of work and unable to identify disease trends, and this contributes to gaps in disease reporting and trend analyses.

Early detection of potential point-source outbreaks is of particular importance, as the detection and removal of the offending source is essential for control (15, 16). Traditional investigation of the intra-species relatedness of diarrhoeal pathogens such as *Shigella* relies on various typing procedures performed in reference public health laboratories. In jurisdictions where *Shigella* isolates are not routinely transferred to a reference laboratory, isolate viability may be compromised, leading to a loss of time and data that are essential for outbreak investigation.

To address this public health gap, we implemented a decentralized, sentinel-site whole-genome sequencing (WGS) capability within a routine diagnostic laboratory in a tertiary hospital (Hospital IV). Hospital IV routinely receives *Shigella* isolates from several hospitals for identity confirmation and/or serogrouping, and is a site that can perform sentinel surveillance. To contain cost and shorten the turnaround time, the Nanopore MinION sequencer (Oxford Nanopore Technologies, United Kingdom) was chosen for sentinel-site sequencing. We hypothesize that the sentinel-site sequence-based surveillance will reduce the time taken to detect a clonal outbreak of *S. flexneri* and that this strategy may provide for early alerts for public health mitigations.

## Methods

### Microbiological investigations

Stool culture isolates identified in Hospital I and Hospital II to be *Shigella* species or *Shigella flexneri* were transferred to Hospital IV for identity confirmation and/or serogrouping. Identity was confirmed in Hospital IV using Vitek 2 GN cards (bioMérieux, Marcy-l'Étoile, France), and observation of motility (–), indole production (+/–), and lysine decarboxylase activity (–) (17). *Shigella* serogrouping was performed using a set of commercial *Shigella* antisera (Mast^®^ Assure antiserum Shigella, Mast Group, Bootle, UK). Antimicrobial susceptibility testing was performed using a combination of disk diffusion, Etest (bioMerieux, Marcy-l'Étoile, France) and Vitek^®^ 2 susceptibility testing (bioMerieux, Marcy-l'Étoile, France). Antimicrobial susceptibility tests were performed and interpreted in accordance with the Clinical and Laboratory Standards Institute (CLSI) standards in Hospitals I, II, IV, and V (18). Hospital III used both CDS (19) and CLSI (18) standards for susceptibility testing. Detailed description of the list of antibiotics tested are summarized in Supplementary Table 1. All isolates identified to be *Shigella flexneri* from 10th March 2022 to 13th April 2022 in Hospitals I-V were included in this study.

### Whole-genome sequencing

Three *S. flexneri* isolates from Hospital I, two *S. flexneri* isolates from Hospital II, and one control *Escherichia coli* isolate (ATCC25922) were subjected to DNA extraction using DNeasy Powersoil Pro Kit (Qiagen, Hilden, Germany) according to the manufacturer's instructions. The extracted DNA samples were prepared using the Q20+ Native Barcoding Kit (SQK-NDB112.24), according to the “Ligation sequencing gDNA - native barcoding (SQKNBD112.24)” protocol. Four hundred nanogram of genomic DNA per sample was used directly for DNA repair and end preparation, without any prior shearing. The samples were incubated at 20 and 65°C for 15 and 5 min, respectively, during the DNA repair and end preparation. The incubation periods for native barcode ligation and adaptor ligation were extended to 30 min. Throughout the protocol, samples were mixed by gentle flicking of the microcentrifuge tube, instead of pipetting. AMPure XP beads (Beckman Coulter, USA) incubation on the revolver rotator (Labnet, USA) was extended to 10 min, and samples were eluted at 37°C for 10 min. To maximize the yield of the libraries obtained, 200 ng of pooled samples were used for adaptor ligation. Each prepared library was loaded into a R10.4 MinION flow cell (FLO-MIN112) and sequenced on a MinION Mk1c machine for >16 h. The acquisition of the sequenced reads was carried out using MinKNOW v21.11.6. For each sample, at least 100 × coverage was obtained and used for downstream analysis. Base-calling, demultiplexing and trimming of barcodes and adaptor sequences were carried out *via* the Guppy v6.1.1 Super High Accuracy basecaller (Oxford Nanopore Technologies).

### Sequence data analysis

The trimmed, filtered reads with an average Phred score >10 were assembled with Flye v2.9 (20) and polished twice with medaka v1.6 (Oxford Nanopore Technologies). The resultant draft genomes were used for downstream analysis. The genome data of the five *S. flexneri* isolates were submitted to the GenBank database under the BioProject ID PRJNA841078, with BioSample accessions SAMN28576597, SAMN28576598, SAMN28576599, SAMN28576600, and SAMN28576601.

Parsnp v1.2 (21) was used for phylogenetic analysis. Fifty-three complete *S. flexneri* reference genomes were downloaded from NCBI (22). All genomes with genetic markers for lacY and without ipaH were removed. The remaining 16 complete reference genomes and five query genomes were included in the phylogenetic analysis. Core genome alignment and single-nucleotide polymorphism (SNP) calling for all included genomes were performed against the reference genome NZ_LR213452.1, with Parsnp's PhiPack (23) option (parsnp-x) to identify regions of recombination. A maximum-likelihood phylogenetic tree was constructed with core genome SNPs (RAxML-NG v1.1.0 with 500 bootstrap replicates). Whole-genome average nucleotide identity (ANI) values were calculated using MUMmer 3.0 (24) alignments.

To search for highly similar *S. flexneri* whole-genome sequences in accessible databases, 9,255 *S. flexneri* whole-genome sequences were downloaded from NCBI (accessed on 17th April 2022) (22). MUMmer 3.0 (24) was used for pairwise alignment between the five query genomes and the 9,255 downloaded *S. flexneri* whole-genome sequences. The top 200 genomes with the highest ANI values, averaged across five query genomes, were then included in the subsequent phylogenetic analysis using Parsnp v1.2 (RAxML-NG v1.1.0 with 500 bootstrap replicates).

ShigaTyper (25) was used for *in silico* serotyping with the latest database (11 February 2022). *In silico* multilocus sequence types (MLST) were determined using publicly available tools in Pathogenwatch (https://pathogen.watch/) using the Enterobase Escherichia/Shigella MLST scheme (https://enterobase.readthedocs.io/en/latest/mlst/mlst-legacy-info-ecoli.html). Genetic markers for antimicrobial resistance and virulence factors were identified using AMRFinderPlus v3.10.24 (26).

## Results

### Timeline of *S. flexneri* detection and analysis in five hospitals

**Figure 1:**
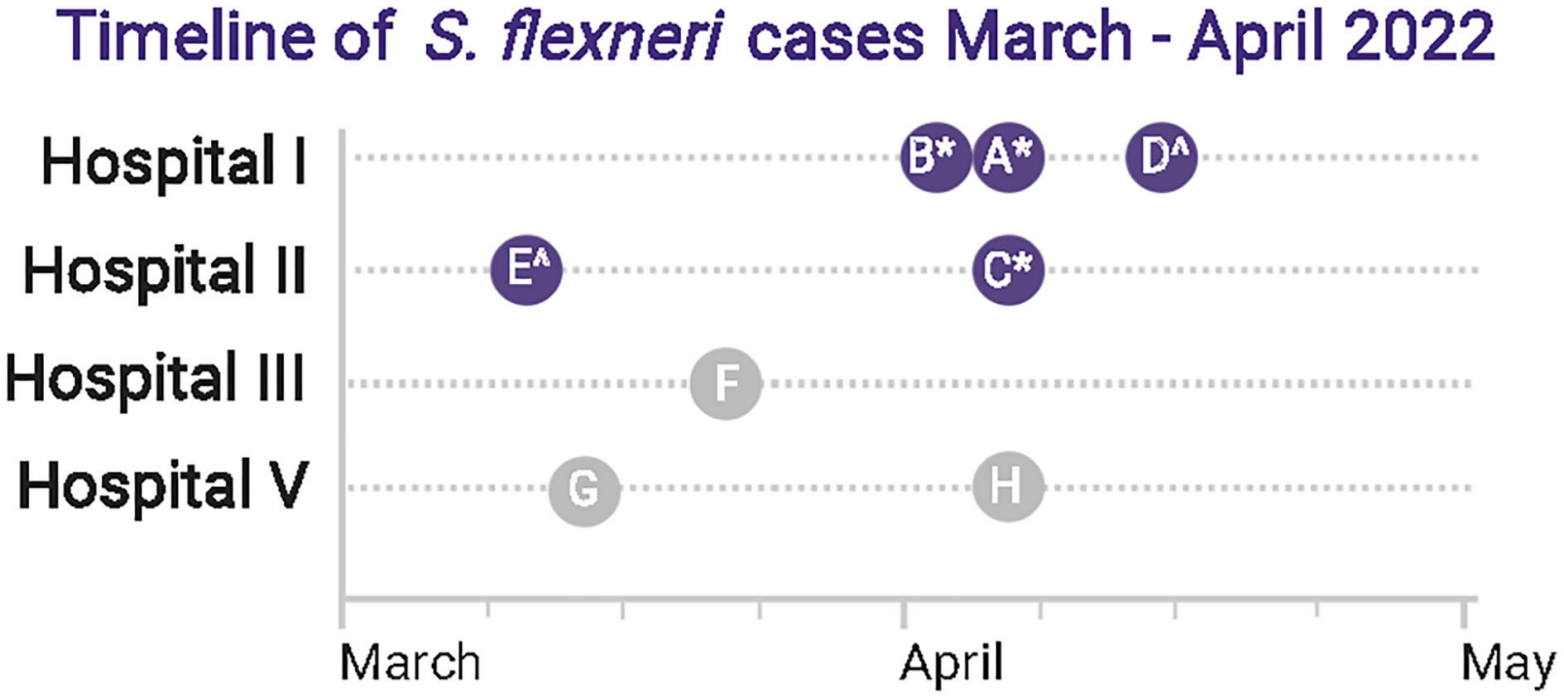
Summary of *Shigella flexneri* detection timeline. There were eight known laboratory-proven *S. flexneri* cases (Cases A–H) in four hospitals within a 5-week period. Five isolates were sequenced in this study (Cases A–E, dark blue circle), among which 3 (^*^) were sequenced near real-time. Two additional isolates were sequenced subsequently (^∧^). Isolates of three cases from Hospitals III and V (Cases F, G, H) had been discarded and were not available for sequence analysis (gray circle). BioRender was used to create this figure.

Figure 1 summarizes the timeline of the detection of eight known *S. flexneri* cases within a 5-week period. Five *S. flexneri* isolates were independently obtained by Hospital I (Cases A, B, and D) and Hospital II (Cases C and E) from 10th March to 13th April 2022. Hospital IV (sentinel-site) received Cases A, B and C for identity confirmation and/or serogrouping within a 2-day period. Cases A, B and C (^*^ in Figure 1) were sequenced near real-time in hospital IV. Once a clonal cluster was suspected based on initial results, communications within the informal hospital laboratory network established that there were at least five additional *S. flexneri* cases from March 2022 to April 2022 among five hospitals. Cases D and E (^∧^ in Figure 1) were sequenced subsequently. The isolates of cases F, G, and H had been discarded and were not available for further analysis.

**Figure 2:**
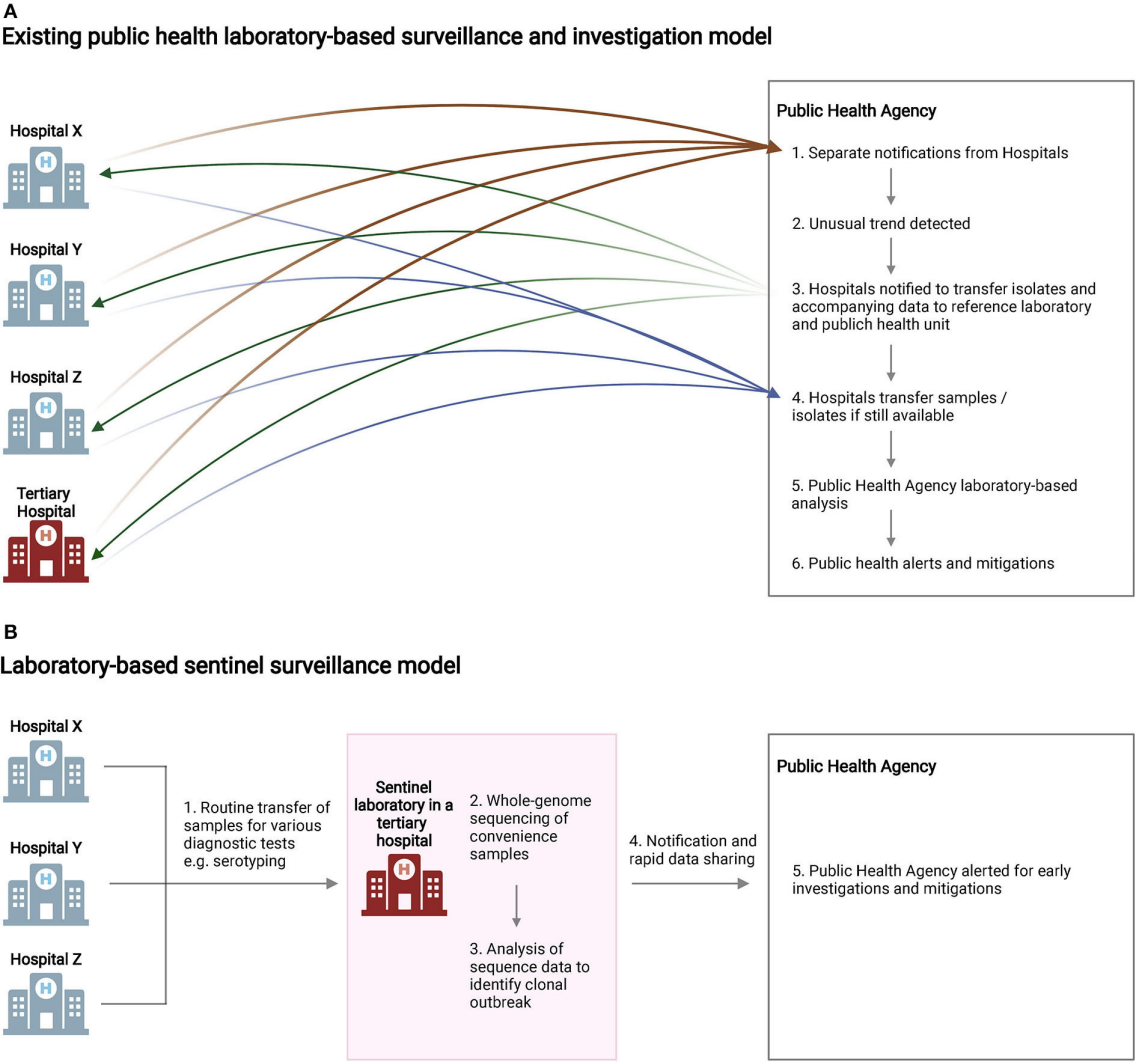
Summary of laboratory-based surveillance models. **(A)** For diseases and isolates not routinely notifiable and transferred to central reference laboratories, delays in outbreak detection and laboratory investigations may occur due to logistical challenges. **(B)** Sentinel surveillance of convenience samples, implemented in the current study, enabled the streamlined detection of clonal isolates of public health importance and provided early alerts for the public health agency. BioRender was used to create this figure.

In total, five *S. flexneri* isolates (Case A–E) were included in the sentinel-site sequence analysis. Antibiograms were retrieved from the remaining three cases (Cases F, G, and H). While not mandatory, the originating laboratories had made individual voluntary notifications to the public health agency at the time of diagnosis for each case (Figure 2A), but the recognition that there was a case cluster occurred at the tertiary hospital (Hospital IV) laboratory.

### Decentralized laboratory-based sentinel surveillance reduced time taken to detect possible clonal outbreak

Current laboratory-based surveillance for pathogens such as *S. flexneri* relies on investigations in central reference laboratories. For diseases and pathogen isolates not routinely notifiable and transferred to the public health reference laboratory, investigations may be delayed due to the time taken for unusual disease trend detection and sample transfers (Figure 2A). Figure 2B illustrates the sentinel surveillance model we used in the current study. An existing network of hospital diagnostic laboratories routinely transfers *Shigella* spp. isolates to a tertiary hospital diagnostic laboratory for consolidated serogrouping and/or identity confirmation. In this study, the same isolates received for diagnostic testing were subjected to WGS within the same laboratory near real-time. The isolates under investigation were found to be genetically closely related within five working days (Day 5) of receipt. On Day 12, the whole-genome comparison results were formally forwarded to the local public health agency. The Singapore Ministry of Health subsequently issued a notification to hospitals (Day 14) to request for archived *Shigella* isolates and any associated data from 2018 to 2022 to be forwarded to the public health agency.

### *S. flexneri* isolates under investigation were genetically closely related

**Figure 3:**
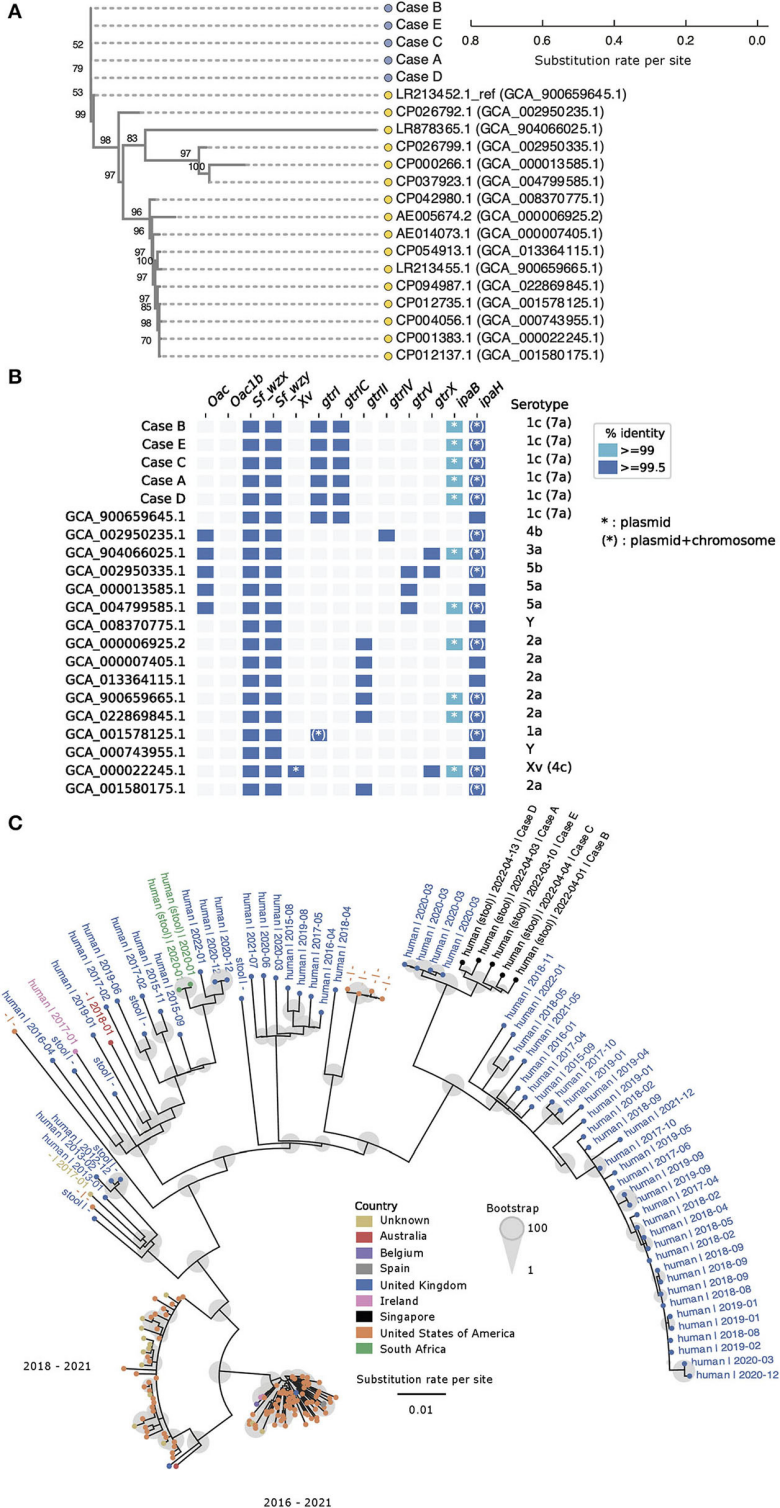
*Shigella flexneri* isolates under investigation are genetically closely related and form a distinct phylogenetic cluster. **(A)** Core genome single nucleotide polymorphisms (SNP) phylogenetic tree generated by Parsnp illustrates phylogenetic relatedness of 16 complete reference genomes (yellow circle) and five query genomes (blue circles). The scale represents substitution rate per site, and the number at each branch indicates the bootstrap support value. **(B)** *in silico* serotyping with ShigaTyper suggests all query isolates are of serotype 1c (7a). The horizontal axis lists ShigaTyper determinants that contributed to the eventual *in silico* serotype (vertical axis). The term “*ipaH*” in this figure represents a conserved 780 bp region of *ipaH* encoding for the C-terminal catalytic domain, which was originally designated as “*ipaH_c*” in the ShigaTyper database. **(C)** Parsnp phylogenetic tree constructed with five Singapore query genomes and 200 most relevant genomes out of 9,255 publicly available *Shigella flexneri* genomes. The scale represents substitution rate per site, and the size of the gray circle at each branch indicates the bootstrap support value. Each genome sequence is represented by a solid circle (∙), and the color of the circle represents the country from which the genome sequence was reported. Genomes that are phylogenetically more closely related to the Singaporean isolates (black circles) are labeled with basic metadata in the following format (sample source | date of sample collection in YYYY-MM). For optimal visualization, genomes that were phylogenetically more distant from the Singaporean isolates were represented by colored solid circles without associated metadata. *Oac, S. flexneri-*specific *O*-acetyltransferase gene marker; Oac1b, *S. flexneri-*specific O-acetyltransferase gene marker specific for serotype 1b and 7b; *Sf_wzx, S. flexneri-*specific O-antigen flippase gene marker; *Sf_wzy, S. flexneri-*specific O-antigen polymerase gene marker; *Xv, S. flexneri-*specific gene marker encoding for protein homolog of LTA synthase family protein; *gtrI*, glucosyltransferase mediating addition of first glucosyl group to the O-antigen backbone in *S. flexneri* serotypes 1 and 7; *gtrIC*, glucosyltransferase mediating addition of second glucosyl group to the O-antigen backbone in *S. flexneri* serotypes 1c (7a); *grtII*, glucosyltransferase of *S. flexneri* serotype 2; *gtrIV*, glucosyltransferase of *Shigella flexneri* serotype 4; *gtrV*, glucosyltransferase of *S. flexneri* serotype 5; *gtrX*, glucosyltransferase of serotypes 2 or X; *ipaB*, invasive plasmid antigen B; *IpaH*, a 780 bp region of the *ipaH* genes encoding the highly conserved C-terminal catalytic domain.

Phylogenetic analysis of the five *S. flexneri* isolates received from two institutions showed that they formed a distinct cluster (Figure 3A). Sixteen other complete *S. flexneri* genomes were included in the core genome phylogenetic analysis using Parsnp (21). A maximum of 20 core genome SNPs differences were observed among the isolates under investigation. The core SNPs matrix is available in Supplementary Table 2. Among the complete chromosomes included in the phylogenetic analysis, the *S. flexneri* strain AUSMDU00008355 (Genbank accession: LR213452.1) was found to be genetically most closely related to the Singapore isolates in this study. AUSMDU00008355 was 172–181 core genome SNPs relative to the Singaporean isolates, and was isolated in 2016 (was made publicly available in 2019) from the stool sample of a symptomatic individual in Australia (27). Pairwise comparisons of the five Singaporean isolates showed that whole-genome average nucleotide identity (ANI) among the five Singaporean isolates was >99.999%, indicating that they were likely to be clonal in nature (Supplementary Figure 1). The whole-genome similarities and SNP matrices of the five query genomes and 16 reference genomes are summarized in Supplementary Figures 1–3.

*In silico* typing was performed to obtain the serotypes and multilocus sequence types (MLST) of the query and reference genomes. *In silico* serotype prediction with ShigaTyper (25) found all five isolates to belong to *S. flexneri* serotype 1c (7a) (Figure 3B). Based on the ShigaTyper reference sequence database, all five query isolates contain the genetic markers for *S. flexneri* specific O-antigen flippase (*Sf_wzx*) and polymerase (*Sf_wzy*). The presence of O-antigen modification genetic markers, namely glucosyltransferases *gtrI* and *gtrIC*, and the absence of O-antigen acetylase 1b (*Oac1b*), gave rise to the *in silico* designation of *S. flexneri* serotype 1c (7a) for all five isolates. Consistent with the species identification and invasive nature of these clinical isolates, the genetic markers for invasive plasmid antigen B (*ipaB*) and invasive plasmid antigen H (*ipaH*) were present in all five isolates. Of note, members of the *ipaH* gene family share a conserved C-terminal catalytic domain, and the term “*ipaH*” used in Figure 3B represents matches to this conserved 780 bp region of *ipaH* (designated as *ipaH_c* in the ShigaTyper database). This conserved region of *ipaH* was detected in the chromosome and the large virulence plasmid of all five genomes under investigation. Ten other complete genomes downloaded from NCBI contained this conserved 780 bp region of *ipaH* in the chromosome and virulence plasmids. The remaining six complete genomes downloaded from NCBI did not contain sufficient information for us to delineate if the conserved 780 bp region of *ipaH* was found in the chromosome or plasmid.

Using the Enterobase *Escherichia/Shigella* MLST scheme, all five isolates were found to belong to the ST245 clonal complex. All five isolates had identical allelic profiles based on gene sequences of seven housekeeping loci (adenylate kinase *adk*, fumarate hydratase *fumC*, DNA gyrase *gyrB*, isocitrate/isopropylmalate dehydrogenase *icd*, malate dehydrogenase *mdh*, adenylosuccinate dehydrogenase *purA*, ATP/GTP binding motif *recA*). The full allelic profiles of all isolates under investigation and reference genomes are summarized in Supplementary Table 3.

To search for other highly similar *S. flexneri* genomes, the five Singapore *S. flexneri* genomes were compared to 9,255 downloaded whole-genome sequences. Among these, 200 public whole-genome sequences with the highest ANI to the five query genomes were selected for subsequently phylogenetic analysis. The five Singapore *S. flexneri* isolates formed a distinct cluster and were found to be most closely related (ANI ≥99.999%, Supplementary Table 4) to a cluster of four assemblies submitted by Public Health England (currently known as the UK Health Security Agency; GCA_013457635.1, GCA_013457815.1, GCA_013457355.10 and GCA_013455205.1; Figure 3C, Supplementary Table 4). All four samples were collected from individuals in the United Kingdom in March 2020 and the sequences were submitted as part of the routine surveillance of *Escherichia coli* and *Shigella* (27).

### *S. flexneri* isolates under investigation contain identical genetic markers for antimicrobial resistance (AMR) and virulence factors

**Figure 4:**
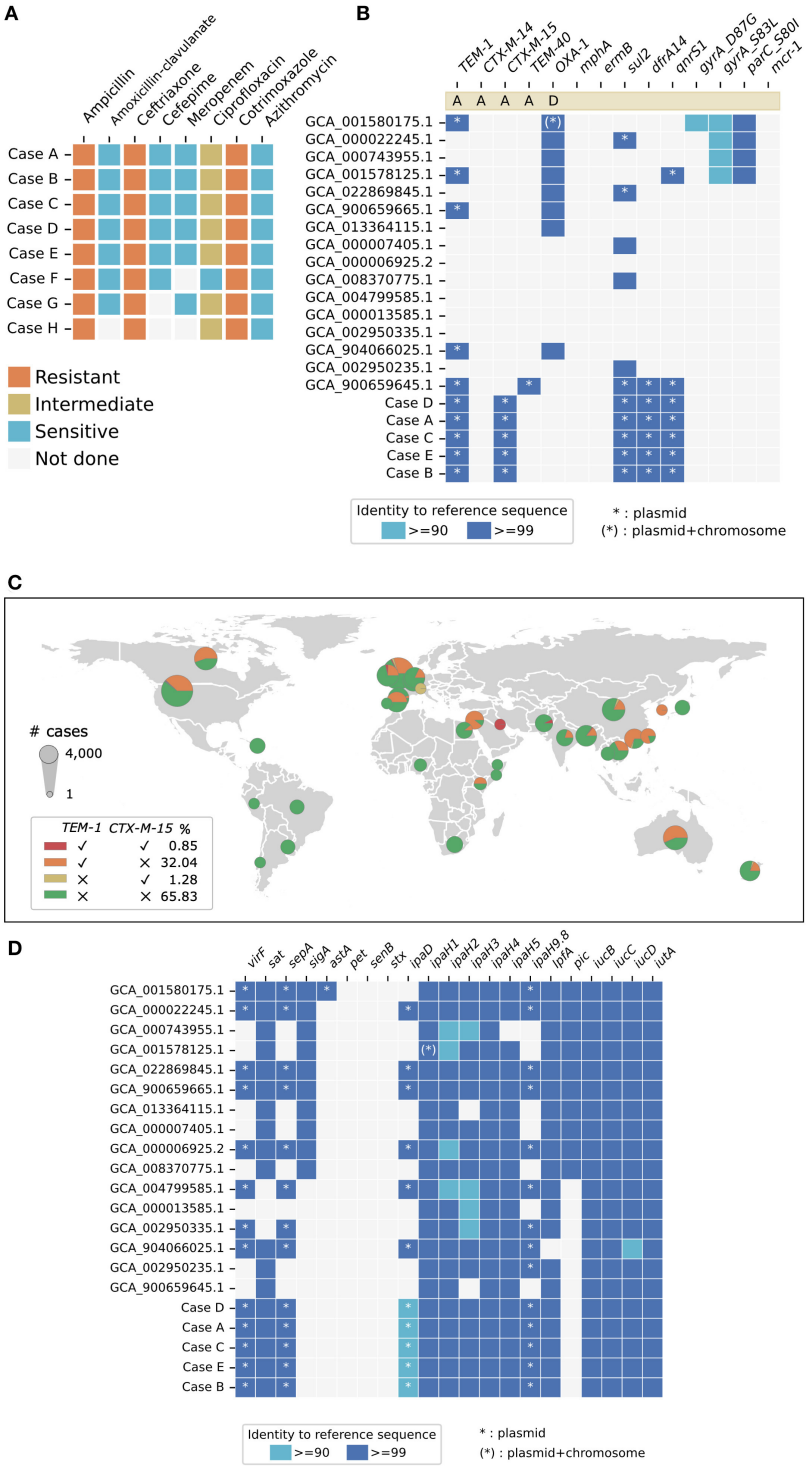
Summary of antimicrobial resistance and virulence markers. **(A)** Cases A–E had identical phenotypic antimicrobial susceptibility profiles. All isolates were resistant to ceftriaxone and had intermediate resistance to ciprofloxacin. The antibiograms for Case F–H were retrieved from the laboratory information systems of Hospital III (Case F) and Hospital V (Case G and H), as the isolates had been discarded and were not available for sequencing. **(B)** Cases A–E had identical genotypic antimicrobial susceptibility profiles and contained *blaCTX-M-15* and *blaTEM-1*, consistent with the extended-spectrum beta-lactamases (ESBL) phenotype observed. **(C)** Global distribution of *blaTEM-1* and/or *blaCTX-M-15* containing *Shigella flexneri* are based on publicly available data (NCBI Pathogen Detection as of 24 April 2022). 0.85% of all available *S. flexneri* genomes (*n* = 11134) contain both *blaCTX-M-15* and *blaTEM-1*. **(D)** Cases A–E had identical genetic markers for a panel of virulence factors, of which *virF, sepA, astA*, and *ipaH9.8* were encoded on plasmids. Sixteen complete *S. flexneri* reference genomes were included for comparison.

Antimicrobial susceptibility testing showed that Cases A–E were multidrug-resistant (MDR) and had identical categorical AMR profiles based on CLSI M100 (32nd edition) (18) interpretation (Figure 4A). All five isolates were resistant to ceftriaxone, with minimum inhibitory concentration (MIC) values ranging from 16 to 32 mg/L. The isolates also demonstrated resistance to cotrimoxazole and intermediate resistance to ciprofloxacin. Ciprofloxacin Etest showed a MIC of 0.38 mg/L (intermediate) for Cases A–E. Cases F–H had been discarded and no further susceptibility testing could be performed. Of note, Case F had been reported to be susceptible to ciprofloxacin by the originating laboratory, based on the CDS method and interpretation (19). This minor difference in categorical interpretation could have been contributed by the ciprofloxacin MIC value of the strain being close to the CLSI M100 (32nd edition) (18) breakpoint of ≤ 0.25 mg/L. All five isolates were resistant to trimethoprim/sulfamethoxazole (MIC ≥320 mg/L). All isolates available for testing remained susceptible to azithromycin, gentamicin, amikacin, cefepime, ertapenem, and meropenem.

All five of the Singapore isolates carried the genes encoding extended-spectrum beta-lactamase (ESBL) *bla*_CTX−M−15_ and *bla*_TEM−1_, consistent with the ceftriaxone-resistant ESBL phenotype observed (Figure 4B). Genetic markers for resistance to sulphonamides (*sul2*) and folate synthesis inhibitors (*dfrA14*) were identified. The presence of *qnrS1* could have contributed to the borderline intermediate resistance to ciprofloxacin. No *gyrA, gyrB* and *parC* mutations associated with quinolone resistance were detected. Genetic determinants conferring resistance to azithromycin, aminoglycosides and tetracycline were not found. Phenotypic and genotypic AMR profiles were therefore concordant for the panel of antimicrobials tested. We looked for, but did not find other AMR genes of significant public health concern, such as *mcr-1* encoding resistance to colistin (28) or genes encoding carbapenemases (29).

The co-existence of the ESBL genes *bla*_CTX−M−15_ and *bla*_TEM−1_ in an *S. flexneri* isolate is an uncommon observation. We analyzed 11,143 publicly available *S. flexneri* genomes for the presence of *bla*_CTX−M−15_ and *bla*_TEM−1_ genes (Figure 4C). Among the 11,143 genomes included in the analysis, 3,665 (32.89%) carry *bla*_TEM−1_ and 238 (2.14%) carry *bla*_CTX−M−15_. There were 95 (0.85%) *S. flexneri* genomes known to be carrying both *bla*_CTX−M−15_ and *bla*_TEM−1_ globally. Out of these 95 ESBL *S. flexneri* genomes, 86 (90.53%) were reported from three high-resource locations, namely the United Kingdom (*n* = 46), Ireland (*n* = 3), and the USA (*n* = 37). Three of the remaining nine ESBL genomes were reported from Bangladesh (*n* = 1), Kuwait (*n* = 1), and Pakistan (*n* = 1). The remainder of the genomes (*n* = 6) did not have associated geographical location data available for analysis. Four of these *bla*_CTX−M−15_*-* and *bla*_TEM−1_*-*carrying genomes (GCA_013457635.1, GCA_013457815.1, GCA_013457355.10, and GCA_013455205.1) were the same genomes identified to have ≥99.99% ANI and cluster most closely with the Singapore *S. flexneri* isolate (Figure 3C).

*In silico* whole-genome sequence analysis identified the same list of chromosomal and plasmid-mediated virulence genes in all five of the Singapore *S. flexneri* isolates (Figure 4D). *VirF*, encoding the master activator necessary for invasion and pathogenicity (30), was found on the large 220kb virulence plasmid, pINV. The virulence gene *ipaH9.8* was found on the plasmid, while *ipaH1, ipaH2, ipaH3, ipaH4* and *ipaH5* were found on the chromosome. The genomes also possess virulence genes responsible for adhesion and cytotoxicity, namely, long polar fimbriae (*lpfA*), secreted autotransporter toxin (*sat*), *Shigella* extracellular protein A (*sepA*). Virulence genes related to enterotoxin production, such as *sigA, pet, senB*, and *stx*, were notably absent in these isolates. Sixteen complete *S. flexneri* genomes downloaded from NCBI which were bioinformatically confirmed to be *S. flexneri* (found to contain genetic marker(s) for *ipaH* but not genetic marker for *lacY*) were included in the analysis. Among these downloaded genomes, eight genomes notably did not contain *virF*, and seven and nine genomes did not contain *ipaH9.8* and *ipaD*, respectively. The lack of consistent detection of these virulence plasmid-mediate genes suggest that existing complete genome data available on NCBI may not reliably detect plasmid-mediate genes. This inconsistency could be due to the loss of the virulence plasmid due to processes related to laboratory handling, or due to limitations related to short-read whole genome sequencing and genome assembly. Nonetheless, due to the limited number of confirmed *S. flexneri* complete genomes (*n* = 16) available, we included all sixteen genomes in the analysis to capture the diversity found in all available data.

## Discussion

We report here the rapid detection of a cluster of clonal MDR *S. flexneri* in Singapore, and the first report of MDR *S. flexneri* serotype 1c (7a), identified using a laboratory-based sentinel surveillance model. Sentinel sequence-based surveillance integrates lower-cost near real-time sequence-based surveillance with routine diagnostic workflow, enabling early detection of possible clonal clusters. The rapid generation of sequence data is particularly advantageous for pathogens and diseases not routinely monitored by public health agencies, as the detection of such outbreaks is likely to be challenging. Curated, portable sequence data generated from sentinel surveillance sites may provide resources for early alerts for public health investigations and interventions.

We demonstrated the clonality of *S. flexneri* isolates in several ways. Firstly, the genomes under investigation were compared to 16 other complete reference *S. flexneri* genomes. Phylogenetic analysis showed that all five genomes belong to the same phylogenetic branch, with a maximum of 20 core genome SNP difference among them (Figure 3A). Secondly, phylogenetic analysis was performed for the five genomes under investigation and 200 publicly available *S. flexneri* whole-genome sequences which were found to be most similar to the query genomes (ANI ≥99.99%). The five genomes under investigation belong to the same distinct phylogenetic branch (Figure 3C). Thirdly, *in silico* typing was performed using ShigaTyper and the Enterobase *Escherichia/Shigella* MLST scheme, and all five isolates were found to belong to serotype 1c (7a) and the ST245 clonal complex, respectively (Figure 3B). Lastly, analysis of AMR and virulence genes showed that all five isolates had identical AMR and virulence gene profiles, which were found to differ from the most closely related publicly available genomes (Figures 4B,D).

This distinct cluster of MDR *S. flexneri* serotype 1c (7a) isolates thus most likely represents a previously unreported clonal source of *S. flexneri* infection. *Shigella flexneri* serotype 1c (7a) was first described in Bangladesh in 1988 (31) and then became widely reported in multiple geographical regions (8, 32–34). Subsequent core genes phylogenetic analysis suggested that there may be two distinct *S. flexneri* 1c lineages, one which originated from ancestral serotype 1a and the other from ancestral serotype 1b (35). Previously reported *S. flexneri* 1c isolates were found to harbor multiple AMR genes, including various extended-spectrum beta-lactamases (35, 36). *Shigella flexneri* serotype 1c (7a) containing *bla*_CTX−M−15_ and *bla*_TEM−1_, however, remains relatively uncommon based on 11,143 publicly available genomes included in our analysis (Figure 4C). To our knowledge, there have been no prior reports of clonal outbreaks caused by *S. flexneri* serotype 1c (7a) containing *bla*_CTX−M−15_ and *bla*_TEM−1_.

The rise in AMR in *S. flexneri* is a public health concern. Current therapeutic guidelines advocate azithromycin, ciprofloxacin or ceftriaxone as first-line therapies, and trimethoprim-sulfamethoxazole or ampicillin as second-line therapies if the isolates are tested susceptible (37). Ciprofloxacin, however, should only be used if the MIC is lower than 0.12 μg/ml, as MIC 0.12 μg/ml or higher may be associated with the presence of a quinolone resistance gene (38). The ciprofloxacin MIC for the isolates described in this study ranged from 0.25 to 0.38 μg/mL, making ciprofloxacin an unfavorable therapeutic option despite the categorical interpretations based on CLSI M100 (18). Therefore, the isolates described in this study cannot be optimally treated with four of the five recommended antibiotics (ampicillin, trimethoprim-sulfamethoxazole, ceftriaxone, and ciprofloxacin), leaving azithromycin as the only therapeutic option.

With increasing reports of MDR and azithromycin-resistant *S. flexneri* (33, 39–45), as well as extensively drug-resistant *S. sonnei* (46, 47), early detection of shigellosis outbreaks carries both therapeutic and public health importance. Current genome databases such as NCBI Pathogen Detection (https://www.ncbi.nlm.nih.gov/pathogens/) facilitate outbreak investigation and resolution by matching pathogen genomes from diverse sources and geographical locations (48). However, the success of this approach is dependent on the availability of genome sequence data from all domains within the One Health framework (49), as well as meticulous standardization of whole-genome sequence data and accompanying metadata, which enables accurate comparisons and source-tracking. In this study, we analyzed 11,134 publicly available *S. flexneri* genomes and 16 complete reference genomes along with our isolates. None of the genomes clustered with our isolates based on our phylogenetic analysis. The most closely related genomes (Figure 3C) were obtained from human clinical samples in the United Kingdom, which were sequenced as part of surveillance. No additional data were available for further investigation or source tracking.

There are several limitations to this study. Firstly, no attempt was made to collect epidemiological or clinical data. Epidemiological and clinical investigations are important parts of traditional outbreak investigations. However, the sentinel sequence-based surveillance model we implemented focused on objective whole-genome sequence data, which is less prone to subjective interpretations, such as patients' recall of dietary history. The outbreak isolate sequence and relatedness data alone are sufficient to constitute an alert for further epidemiological and public health investigations by the appropriate agencies. Secondly, the sentinel site did not serve all hospitals in Singapore. The sentinel site routinely receives *Shigella* isolates from three district hospitals and two community hospitals. Nonetheless, we were able to analyze five isolates from two geographically distinct district hospitals, as well as obtained information on additional cases from two other hospitals. Thirdly, we used the nanopore platform for rapid whole-genome sequencing. A major criticism of the nanopore platform has been its base-level accuracy. We have mitigated this risk by achieving a high sequencing depth (>100 × coverage), as well as using the super high accuracy basecaller and polishing the draft genome.

In conclusion, we were able to detect a clonal cluster of MDR *S. flexneri* near real-time by sentinel sequence-based surveillance. Compared to the traditional central reference laboratory structure, this strategy saved time and resources by extending existing routine laboratory workflows. We were able to alert the public health agency of a possible clonal cluster within an actionable timeframe. As jurisdictions work to improve pandemic preparedness, considerations should be made to develop a network and infrastructure for sentinel sequence-based surveillance, which has the flexibility to be rapidly deployed during sporadic outbreaks, epidemics, and pandemics (50).

## Data availability statement

The original contributions presented in the study are included in the article/Supplementary materials, further inquiries can be directed to the corresponding author. The genome data of the five *S. flexneri* isolates were submitted to the GenBank database under the BioProject ID PRJNA841078, with BioSample accessions SAMN28576597, SAMN28576598, SAMN28576599, SAMN28576600, and SAMN28576601.

## Author contributions

KK and CS conceptualized, designed, and planned the project. JChu, KL, WH, ST, and DC planned and performed wet-lab experiments with KK's and CS's supervision. HY performed computational analysis with CS's supervision. KG, ST, TN, MM, JChi, YT, JS, and TK provided clinical samples and clinical expert opinion. KK, HY, and CS wrote the manuscript with inputs from all authors. NN provided feedback on computational and wet-lab experiments, as well as manuscript. All authors contributed to the article and approved the submitted version.

## Funding

KK is supported by the Singapore National Medical Research Council Research Training Fellowship. This work is supported by funding's from Singapore General Hospital, Chiang Mai University and A^*^STAR.

## Conflict of interest

The authors declare that the research was conducted in the absence of any commercial or financial relationships that could be construed as a potential conflict of interest.

## Publisher's note

All claims expressed in this article are solely those of the authors and do not necessarily represent those of their affiliated organizations, or those of the publisher, the editors and the reviewers. Any product that may be evaluated in this article, or claim that may be made by its manufacturer, is not guaranteed or endorsed by the publisher.
