# Supplementary material for: Clonal serotype 1c multidrug-resistant Shigella flexneri detected in multiple institutions by sentinel-site sequencing
Source: Front Med (Lausanne). 2022 Aug 1;9:964640. doi: 10.3389/fmed.2022.964640 (PMC9376355; doi:10.3389/fmed.2022.964640)
Supplement: Supplementary file 1 [file Table_1.DOCX]

**Supplementary Table 1**. Materials used for antibiotic susceptibility testing

**Table 1A** Summary of methods used for antibiotic susceptibility testing

| Isolate | Susceptibility test method and materials |
| --- | --- |
| A | Vitek® 2 system* (bioMerieux, Marcy-l'Étoile, France) and *Vitek® 2 AST-N356 card (bioMerieux, Durham, USA) was used for susceptibility testing. The antibiotics included in the card and the respective concentrations are listed in **Table 1B** below.  ETEST^ (bioMerieux, Marcy-l'Étoile, France) used are listed in **Table 1C** below. |
| B |  |
| C |  |
| D |  |
| E |  |
| F | Disk diffusion method was used, and the antibiotic disks (Thermo Fisher Scientific Australia, Scoresby, VIC, Australia) and respective potency are listed in **Table 1D**. Ciprofloxacin ETEST (bioMerieux, Marcy-l'Étoile, France) was used and the details are available in **Table 1C**. The primary testing was done according to the CDS standard ([http://cdstest.net](http://cdstest.net/)). |
| G | Disk diffusion method was used, and the antibiotic disks (Oxoid / ThermoFisher Scientific, Basingstoke, UK) and respective potency are listed in **Table 1E**. Azithromycin ETEST (bioMerieux, Marcy-l'Étoile, France) was used and the details are available in **Table 1C**. |
| H |  |

**Table 1B** List of antibiotics included in Vitek® 2 susceptibility testing

| *Vitek® 2 AST-N356 card antibiotic | Concentration tested (µg/mL) |
| --- | --- |
| Amikacin AN | 8, 16, 64 |
| Amoxicillin-clavulanate AMC | 4/2, 16/8, 32/16 |
| Amoxicillin AM | 4, 8, 32 |
| Aztreonam ATM | 2, 8, 32 |
| Cefazolin CZ | 4,16, 64 |
| Cefepime FEP | 0.25, 1, 4, 16, 32 |
| Cefoxitin FOX | 8, 16, 32 |
| Ceftazidime CAZ | 0.25, 1, 2, 8, 32 |
| Ceftriaxone CRO | 0.12, 0.25, 1, 4,16 |
| Ciprofloxacin CIP | 0.5, 2, 4 |
| Ertapenem ETP | 0.03, 0.12, 0.5, 2 |
| Fosfomycin FOS | 8, 16, 32 |
| Gentamicin GM | 4, 16, 32 |
| Meropenem MEM | 0.5, 2, 6, 12 |
| Nitrofurantoin FT | 16, 32, 64 |
| Piperacillin/Tazobactam TZP | 2/4, 8/4, 24/4, 32/4, 32/8, 48/8 |
| Trimethoprim/Sulfamethoxazole SXT | 1/19, 4/76,16/304 |

**Table 1C** List of ETEST® used for susceptibility testing

| ^ETEST® | Concentration tested (µg/mL) |
| --- | --- |
| Ciprofloxacin CI (REF 412311) | 0.002 - 32 |
| Azithromycin AZ (REF 412257) | 0.016 - 256 |

**Table 1D** List of antibiotics used for disk diffusion susceptibility testing of Case F

| Antibiotic disk | Disk potency (µg) |
| --- | --- |
| Ampicillin AMP | 25 |
| Amoxicillin-clavulanate AMC | 40/20 |
| Ceftriaxone CRO | 5 |
| Cefotaxime CTX | 5 |
| Ciprofloxacin CIP | 2.5 |
| Trimethoprim/Sulfamethoxazole SXT | 1.25/23.75 |
| Azithromycin AZM | 15 |

**Table 1E** List of antibiotics used for disk diffusion susceptibility testing of Case G and H

| Antibiotic disk | Disk potency (µg) |
| --- | --- |
| Ampicillin | 10 |
| Amikacin | 30 |
| Amoxicillin-clavulanate | 30 |
| Ceftazidime | 30 |
| Cefoxitin | 30 |
| Ciprofloxacin | 5 |
| Ceftriaxone | 30 |
| Cefotaxime | 30 |
| Cefuroxime | 30 |
| Cefazolin | 30 |
| Ertapenem | 10 |
| Cefepime | 30 |
| Gentamicin | 10 |
| Meropenem | 10 |
| Ampicillin-sulbactam | 20 |
| Trimethoprim/Sulfamethoxazole SXT | 25 |
| Piperacillin/Tazobactam | 110 |
