# Supplementary figures and images for: Clonal serotype 1c multidrug-resistant Shigella flexneri detected in multiple institutions by sentinel-site sequencing
Source: Front Med (Lausanne). 2022 Aug 1;9:964640. doi: 10.3389/fmed.2022.964640 (PMC9376355; doi:10.3389/fmed.2022.964640)

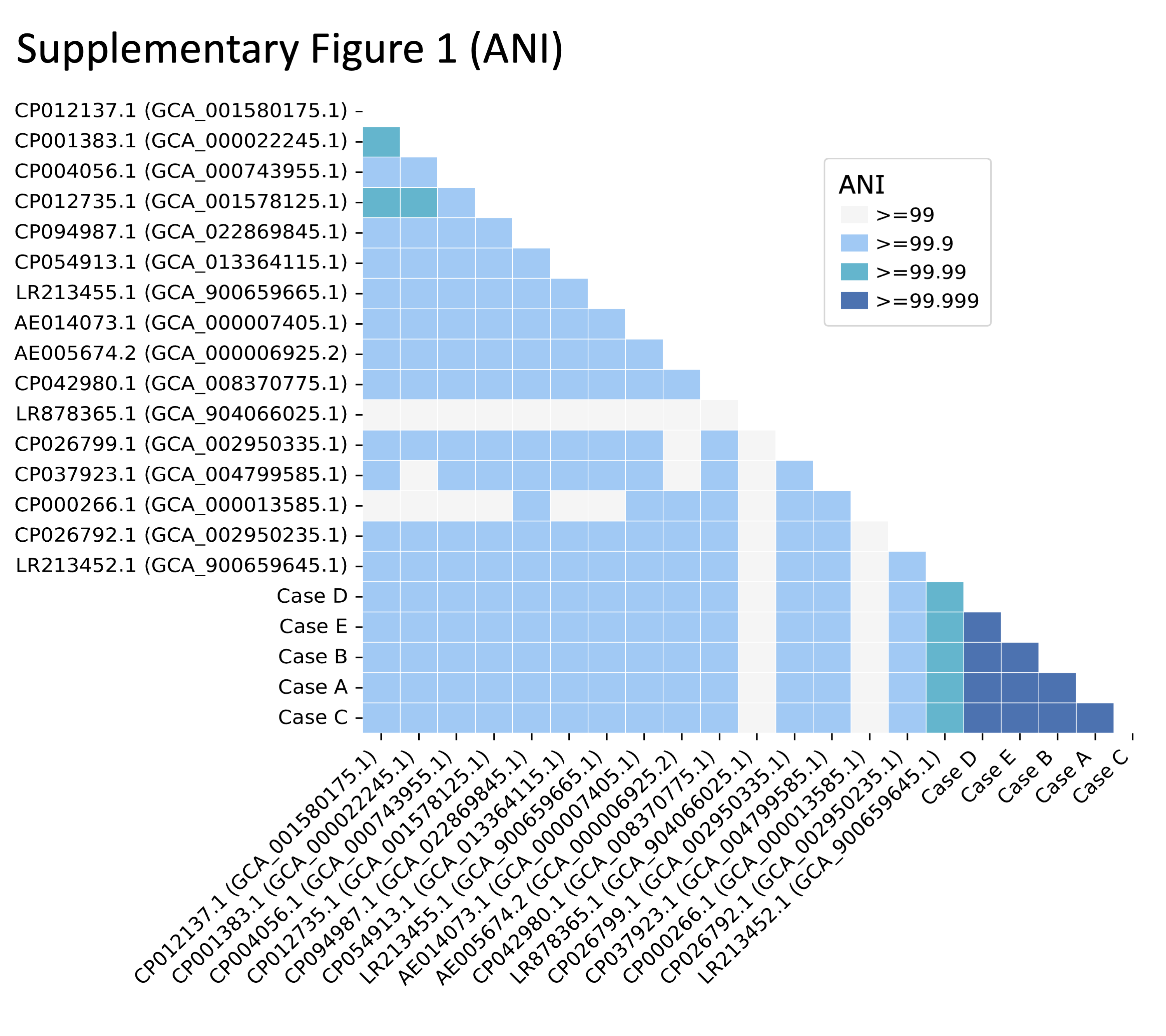

Supplement: Supplementary file 5 [file Image_1.TIFF]

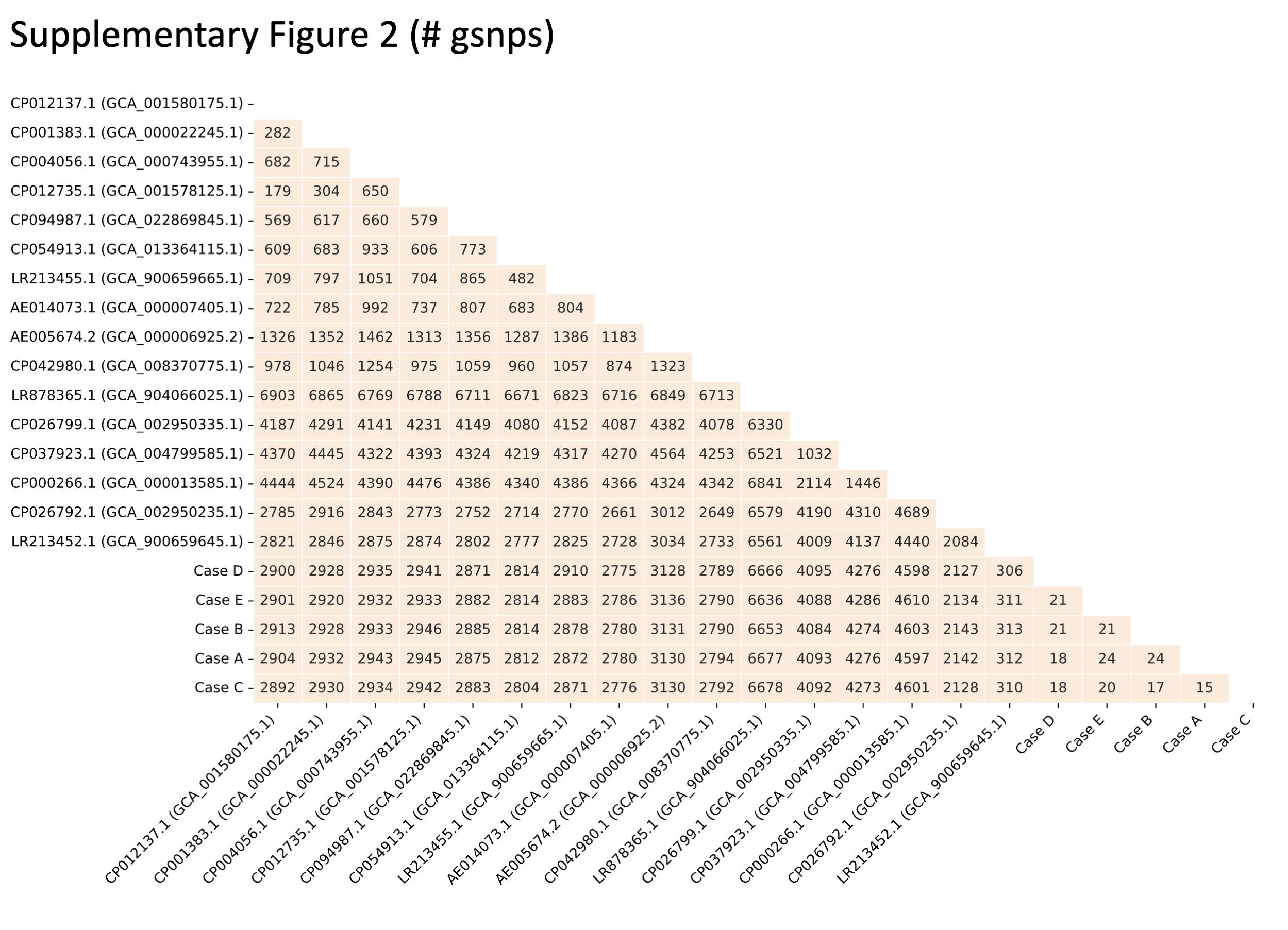

Supplement: Supplementary file 6 [file Image_2.TIFF]

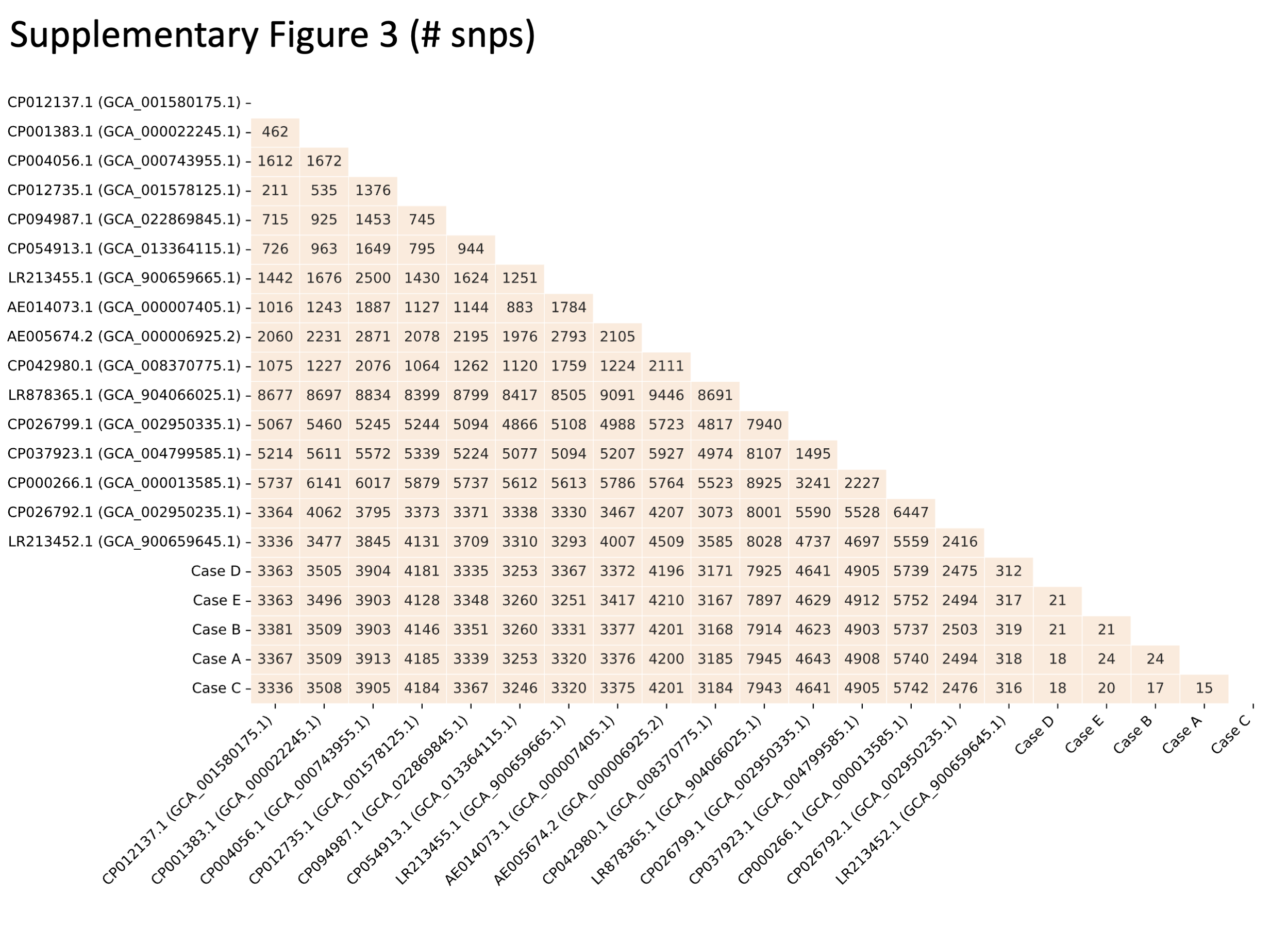

Supplement: Supplementary file 7 [file Image_3.TIFF]
